# The Interplay between Oxidative Phosphorylation and Glycolysis as a Potential Marker of Bladder Cancer Progression

**DOI:** 10.3390/ijms21218107

**Published:** 2020-10-30

**Authors:** Greta Petrella, Giorgia Ciufolini, Riccardo Vago, Daniel Oscar Cicero

**Affiliations:** 1 Dipartimento di Scienze e Tecnologie Chimiche, Università di Roma “Tor Vergata”, 00133 Rome, Italy; petrella@scienze.uniroma2.it (G.P.); giorgia.ciufolini@live.com (G.C.); 2 Urological Research Institute, IRCCS Ospedale San Rafaele, 20132 Milan, Italy; 3 Faculty of Medicine and Surgery, Università Vita-Salute San Rafaele, 20132 Milan, Italy

**Keywords:** urothelial bladder cancer, nuclear magnetic resonance, exo-metabolomics, glycolysis, oxidative phosphorylation, cancer progression

## Abstract

Urothelial bladder cancer (UBC) is the most common tumor of the urinary system. One of the biggest problems related to this disease is the lack of markers that can anticipate the progression of the cancer. Genomics and transcriptomics have greatly improved the prediction of risk of recurrence and progression. Further progress can be expected including information from other omics sciences such as metabolomics. In this study, we used ^1^H-NMR to characterize the intake of nutrients and the excretion of products in the extracellular medium of three UBC cell lines, which are representatives of low-grade tumors, RT4, high-grade, 5637, and a cell line that shares genotypic features with both, RT112. We have observed that RT4 cells show an activated oxidative phosphorylation, 5637 cells depend mostly on glycolysis to grow, while RT112 cells show a mixed metabolic state. Our results reveal the relative importance of glycolysis and oxidative phosphorylation in the growth and maintenance of different UBC cell lines, and the relationship with their genomic signatures. They suggest that cell lines associated with a low risk of progression present an activated oxidative metabolic state, while those associated with a high risk present a non-oxidative state and high glycolytic activity.

## 1. Introduction

Bladder cancer, of which urothelial bladder carcinoma (UBC) represents 90% of all diagnosed cases [1], is the most common cancer of the urinary tract and annual worldwide estimates amount to 429,000 new cases and 165,000 deaths [2]. UBC is divided into low-grade non-muscular invasive bladder cancer (NMIBC) and high-grade muscular invasive bladder cancer (MIBC) according to the European Association of Urology (EAU) guidelines. Early diagnosis of bladder cancer has a better prognosis with a 10-year disease-free survival of 86% for NMIBC. However, due to the absence of reliable prognostic markers, high recurrence rates and risk of progression to MIBC phenotype for high-risk patients are frequently observed [3]. This makes UBC treatment one of the most expensive per patient among all types of cancer [4] and emphasizes the urgent need to understand the molecular basis of UBC progression.

One possible strategy for understanding at the metabolic level what happens during UBC progression is to use urothelial bladder cancer cell lines (UBCcls). They constitute very useful and simple models of the disease, although it is always important to consider that they cannot fully reflect the genetic and phenotypic diversity of primary tumors. In order to correctly extrapolate the results obtained with the cells to predict what is happening at the tumor level, it is necessary to carefully choose those lines that can best explain the specific biological question. Traditionally, UBCcls were classified taking into account the pathological grade, the staging system, and the clinical prognosis of the tumors from which they were isolated. However, often tumors with similar pathologies exhibit different biological behaviors, therefore making the use of additional information such as genomic-based molecular classification indispensable [5]. Using a comprehensive classification based on these markers [6], we selected the RT4 and 5637 lines associated to tumors with low- and high-risk of progression, respectively. In addition, we also chose the RT112 line, which presents common characteristics with the other two and represents a kind of “missing link” in the progression of UBC.

In this study, we have metabolically characterized these three cell lines, with the purpose of understanding if the genotypes that were associated to different risks of recurrence and progression [6] determine sufficiently different phenotypes in order to provide new prognostic markers for in vivo analysis. For this purpose, we have used ^1^H-NMR to measure the exo-metabolic profile of the three UBCcls. Unlike traditional cell metabolomics, which measures the relative concentrations of metabolites within the cell, exo-metabolomics investigates variations in concentrations in the culture medium and provides information on the nutrient consumption and excretion of metabolic products. It probably represents the simplest method to understand the role of major pathways in cell line metabolism.

## 2. Results

In this work, we have compared the exo-metabolome of three different UBCcls: 5637, RT112, and RT4. In our conditions, these three cell lines show different grow rates: 5637 and RT112 cells show a faster proliferation (μ = 0.03 h^−1^) than RT4 cells (μ = 0.02 h^−1^) (Figure S1). In a typical experiment, cells were grown in RPMI culture medium for 1, 2, or 3 days in separated containers, and the experiment was repeated 4 to 5 times to have enough replicates for statistical analysis.

Using ^1^H-NMR spectroscopy, we have followed the daily variation of the concentration in the extracellular medium of 35 metabolites. They included amino acids (66%), organic acids and derivatives (17%), carbohydrates (6%), and others (11%) (Figure S2). The assignment of the metabolite signals in the ^1^H-NMR spectra was confirmed using two-dimensional experiments, such as ^1^H-^1^H TOCSY and ^1^H-^13^C HSQC experiments. The list of chemical shifts used to uniquely identify all metabolites can be found in the supplementary material (Table S1).

**Figure 1:**
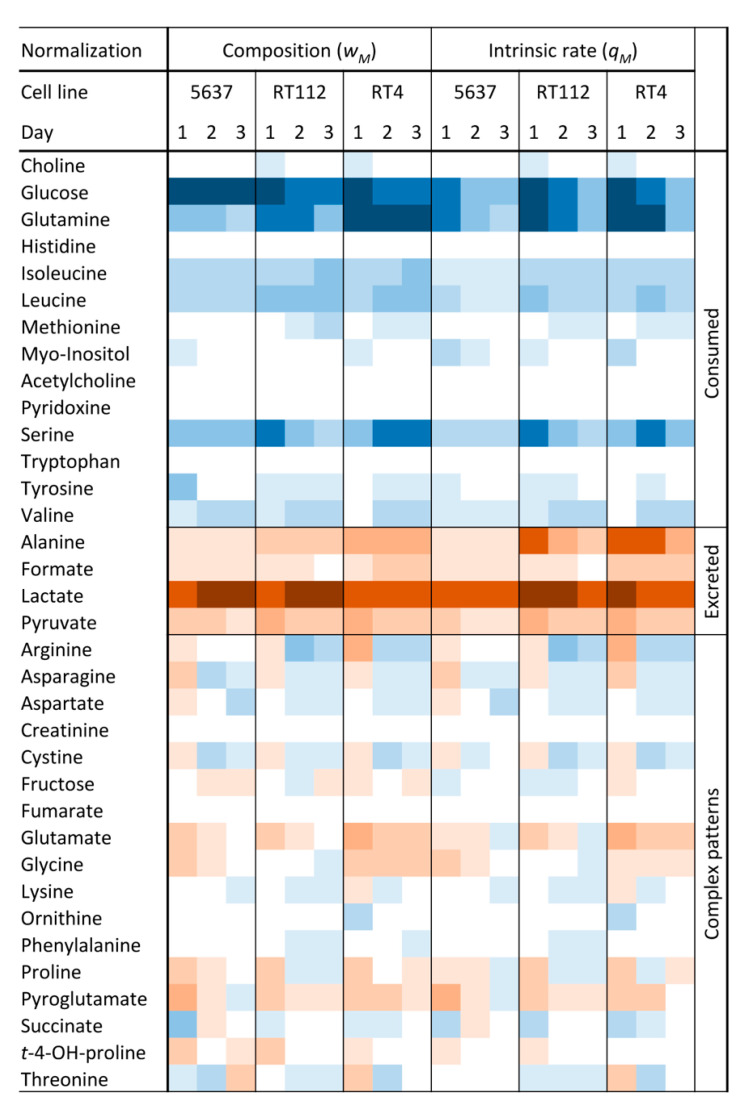
Heatmap showing the daily variations in metabolite concentrations for the three urothelial bladder cancer cell lines (UBCcls). Variations normalized using both cell growth or total composition are shown. Light blue and brown colors are used for consumption and excretion, respectively. Variations increase from the light to the dark colors.

Before the data analysis, two types of normalizations were applied (see S.M.). In the first one, the rate of variation of a metabolite is divided by the rate of cell growth, obtaining the specific rate of variation, *q_M_*, in pmol/cell. The second normalization takes into account the relative molar weight of the variation of a metabolite with respect to the appropriate sum of all other compounds that are consumed (*w_S_*) or excreted (*w_P_*). These two normalization schemes help to understand the role of a compound in the cell metabolism from two different points of view. *q_M_* measures the degree of upregulation of the exchange between the compound and the extracellular medium or of the metabolic pathway in which it is involved. The *w_S_* value can be interpreted as the weight of a nutrient on the total “diet” of a cell, although it does not necessarily reflect the degree of activation but the relative participation of the related pathway in the total metabolism of the cell. The analysis of the normalized variations shows that 40% of the metabolites were only consumed, 11% were only excreted, and 49% showed a complex pattern (Figure 1). Among the nutrients, glucose shows the highest variation, followed by glutamine, serine, branched-chain amino acids (BCAAs), and arginine. The excretion profile is mainly formed by lactate and alanine, along with small amounts of pyruvate and formate.

Then, a multivariate model was calculated to determine the degree of exo-metabolome differentiation of the three UBCcls including data from all daily variations. The corresponding supervised model (Orthogonal PLS Modeling in the discriminant version (OPLS-DA)) showed a significant separation among the three classes (Figure S3). The variables that mostly contributed to the separation included metabolites belonging to the pyruvate and serine metabolisms, plus arginine, glutamine, and BCAAs. These particular pathways will be examined in detail in the following sections.

### 2.1. Glucose and Lactate are Predominant in the Exo-Metabolomic Profile of 5637 Cells

**Figure 2:**
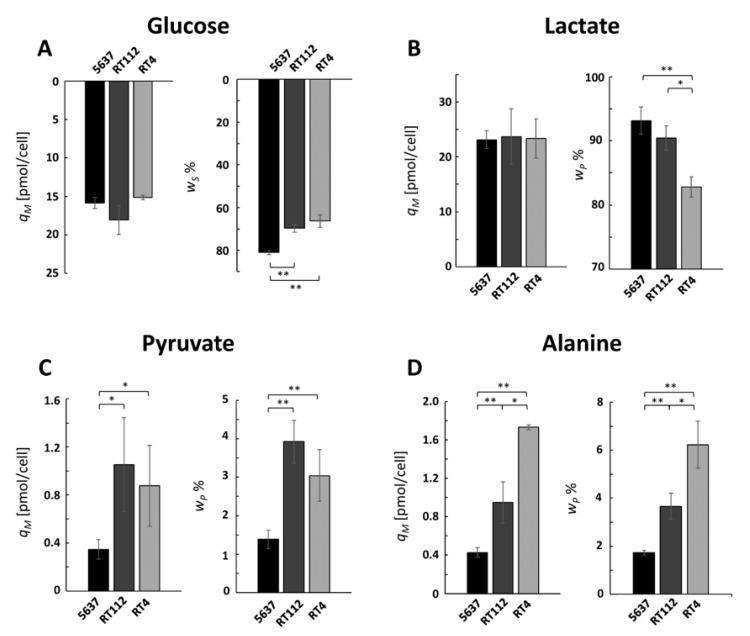
Variations in glucose (**A**), lactate (**B**), pyruvate (**C**), and alanine (**D**) extra-cellular concentrations observed during the third day and normalized by cell growth (left panel) and intake or excretion profile (right panel). * *p* < 0.05; ** *p* < 0.01.

The calculated *q_M_* values for glucose and lactate of the three lines were very similar (Figure 2A,B, left panels). Those of glucose were all around 17 pmol/cel, which is in agreement with the values of 14 and 17 pmol/cel measured for RT4 and TCSSUP cells, respectively [7]. Given that the growth rates of 5637 and RT112 cells are 34% higher than those of RT4 cells, and that the calculated *q_M_* value is the same for all lines, the former consume 34% more glucose per hour than the latter, but the higher consumption is explained exclusively by the faster cellular replication and not by an upregulation of glucose uptake. A similar reasoning can be made for lactate, which is excreted 34% more per hour by lines 5637 and RT112 than by RT4.

The weight of glucose in the diet of the three cell lines is different: it represents 83%, 70%, and 68% of the total diet of cells 5637, RT112, and RT4, respectively (Figure 2A, right panel). As a consequence, also, the weight of lactate in the total excretion is different and represents 90% for 5637 and RT112 cells against only 82% of RT4 cells (Figure 2B, right panels). An analysis of these data shows that glucose consumption and lactate excretion are the most important events in the exchange of compounds between 5637 cells and the extracellular medium, while RT cells show a more varied consumption with a lower glucose weight.

While all cell lines excreted small amounts of pyruvate, cells 5637 did so to a lesser extent (Figure 2C). On the other hand, alanine excretion is higher for RT4 cells (Figure 2D) and shows a very clear trend among the UBCcls, in the order RT4 > RT112 > 5637. These values show that within the 5637 cells, most of the pyruvate finishes its fate converted into lactate, confirming that the consumption of glucose and excretion of lactate dominate the exchange of substances of these cells with the extracellular medium.

### 2.2. Glycolysis is the Most Active Metabolic Pathway in 5637 Cells

The pyruvate produced by glycolysis can either be transformed into lactate in the cytosol or enter the mitochondria where, among other reactions, it can be converted to alanine through a transamination reaction using the amino group of glutamate and producing alpha-ketoglutarate [8,9]. This means that the value of lactate excretion is directly proportional to the degree of glycolysis activity, whereas the degree of alanine excretion can be used as a measure of mitochondrial and OxPhos activities. For this reason, the lactate/alanine ratio is a metabolic measurement of the glycolysis/OxPhos equilibrium.

**Figure 3:**
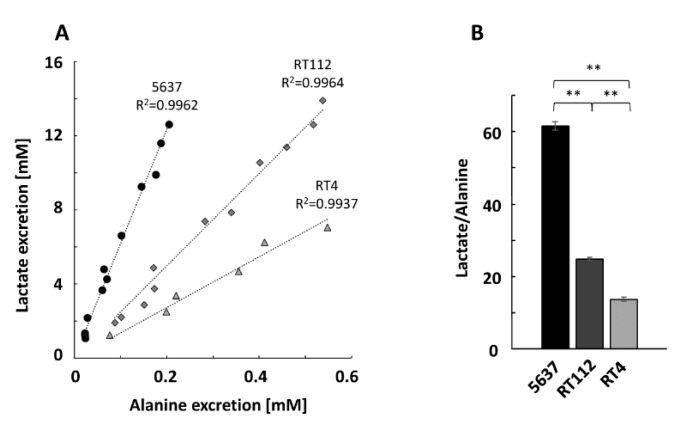
In vitro measurement of the lactate/alanine ratio. (**A**) Correlation graphic between lactate and alanine excretion for the three cell lines: 5637 (circles), RT112 (rhombuses), RT4 (triangles). (**B**) Slope values of the lactate/alanine correlation lines. ** *p* < 0.01.

We have noticed a high and significant correlation between the concentrations of lactate and alanine excreted during the three days by the UBCcls (Figure 3A). The fact that there is a linear relationship indicates that the relative amount of pyruvate used for the synthesis of one or the other metabolite remains constant during the 72 h. From the analysis of the slope values, it is possible to calculate that cells 5637 excrete 62 lactate molecules per alanine molecule, while this value is reduced to 25 and 14 for cells RT112 and RT4, respectively (Figure 3B). These numbers translate very sensitively the balance between non-oxidative and oxidative metabolic states observed so far, correlating a high value of the lactate/alanine index with the preponderant use of glycolysis and a low one with OxPhos. Considering all these data, it is possible to conclude that glycolysis is the major active pathway for the production of ATP and biomass in 5637 cells, is of intermediate importance for RT112 cells, and is of minor importance for RT4 cells.

### 2.3. RT Cells Show an Active Oxidative Metabolism

**Figure 4:**
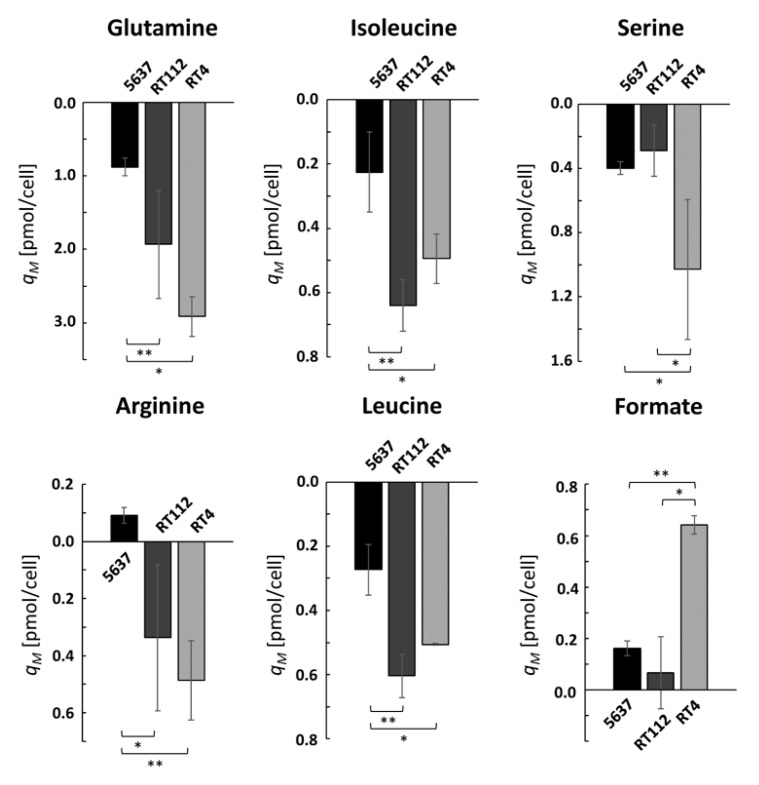
Variations in extracellular concentration of glutamine, isoleucine, arginine serine, leucine, and formate measured during the third day and normalized by cell growth. * *p* < 0.05; ** *p* < 0.01.

RT4 cells show increased consumption of arginine, glutamine, BCAAs, and serine, along with an increased excretion of formate with respect to 5637 cells (Figure 4). All these compounds are related to metabolic pathways that occur inside the mitochondria, require an active oxidative metabolism, and were already found altered in different tumors and cancer cell lines [10,11,12]. RT4 cells show also a higher serine consumption than the other two cell lines, along with a higher excretion of a product of its catabolism, formate, which is a further confirmation of an active mitochondrial activity and active oxidative phosphorylation metabolism (OxPhos) [13]. RT112 cells, on the other hand, show a similar consumption of glutamine and BCAAs with respect to RT4 cells, but serine consumption and glycine and formate excretions are lower. This implies that the activation of the OxPhos metabolism is intermediate between those of RT4 and 5637 cells.

### 2.4. The Risk of Progression Associated with Cell Lines Correlates with the Balance between Glycolysis and Oxphos

**Figure 5:**
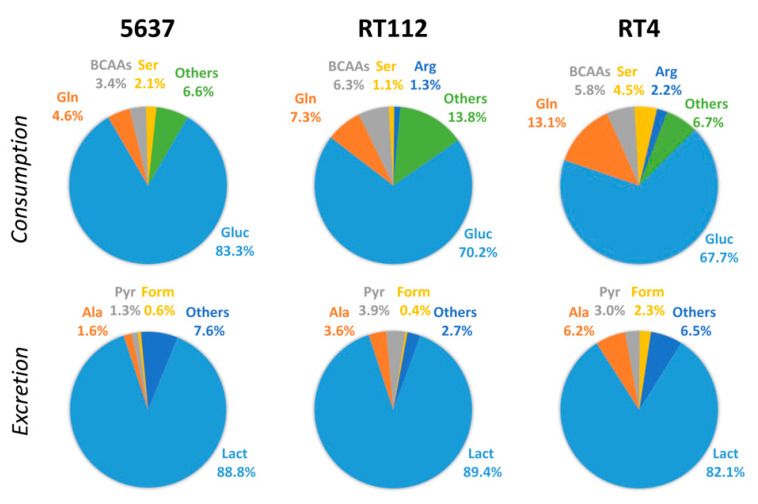
Diet composition (**top** line) and excretion (**bottom** line) of the three cell lines. Abbrevations used: Ala: alanine, Arg: arginine, BCAAs: branched-chain amino acids, Form: formate, Gluc: glucose, Gln: glutamine, Lact: lactate, Pyr: pyruvate, Ser: serine.

The diet and excretion profiles of the three lines are shown in Figure 5, as a summary of all the variations so far described. These graphs clearly reveal the different weights that glucose has in the three diets, decreasing its importance in the 5637–RT112–RT4 series. In contrast, glutamine, BCAAs, and arginine show an opposite trend and are more important in the diet of RT cells. Regarding the excretion profile, lactate shows the same high weight for the two lines with fast growth, 5637 and RT112. The excretion of alanine follows an opposite behavior, in line with the shift of pyruvate metabolism, which is the cause of lower lactate excretion and higher alanine excretion in RT4 cells.

**Figure 6:**
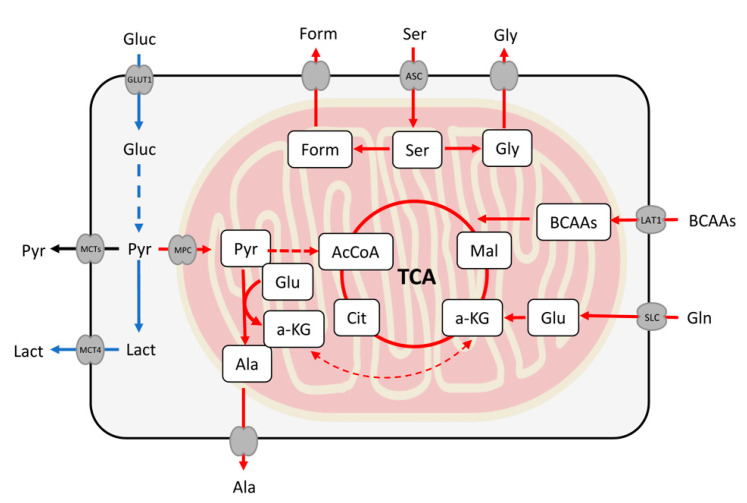
Differently regulated biochemical pathways in the UBC cell lines studied. The blue arrows represent the active metabolism in cells associated with high risk of progression (5637), while the red arrows represent those at low risk (RT4). The RT112 line presents a lower catabolism of serine, which is manifested in a lower excretion of formate and glycine. The abbreviations used are the same as in Figure 5, plus AcCoA: acetyl-CoA, a-KG: alpha-ketoglutarate, Cit: citrate, Glu: glutamate, Gly: glycine, Mal: malate.

Figure 6 shows the different pathways described so far, with the indication of the degree of activity suggested for the different cell lines studied. The interpretation of these results allows us to conclude that 5637 cells, associated to tumors with high risk of progression, consume almost exclusively glucose and excrete lactate, which is an index of a high weight of glycolysis in their metabolism. At the other extreme, RT4 cells, associated with tumors at a low risk of progression, show mitochondrial activity and consequently active OxPhos metabolism. RT112 cells are metabolically located between the other two lines, which is in accordance with their mixed genetic signature.

## 3. Discussion

The main objective of this work was to investigate the connection between the metabolism of UBC cells and the risk of progression of the disease, which ultimately can lead to new prognostic markers based on metabolomics. Genomics has recently made great advances in relating alterations in cellular genes to the propensity to convert NMIBC into MIBC [14]. For example, mutations in *FGFR3* were mostly observed in patients with the lowest risk of progression among those showing NMIBC [15], and *FGFR3*, *PIK3CA*, or *TERT* alterations were not associated with progression [14]. On the contrary, mutations in *TP53* are common in advanced tumors and a key factor that triggers NMIBC progression [14,16]. Considering this information, we have chosen the cell lines RT4 and 5637, which show mutations in *FGFR3* or *TP53* [6], as good representatives of tumor cells with low- and high-risk of progression, respectively. RT112 cells, which show both mutations, represent an intermediate class with an unknown associated risk.

Given the strong evidence of a connection between genotype and risk of UBC progression, we have explored in this work how different the cellular phenotypes associated to the various genomic signatures are, and if metabolomics can add easily accessible markers to improve the predictability of such risk in vivo. To perform this task, we have characterized the exo-metabolome of the three lines using ^1^H-NMR and followed the daily variation of 35 metabolites for 72 h. We have verified that while the *TP53* altered cells 5637 mainly use glycolysis, RT4 cells containing an alteration in *FGFR3* base their energy production on OxPhos. The RT112 cells that present both genomic signatures partially use both metabolic pathways.

The mutation in *TP53* shown by 5637 cells may be closely related to their high glycolytic activity. Cells with p53 knock-down depend more on glycolysis and produce more lactate than wild-type cells [17]. On a molecular level, p53 inhibits PDK2, which is a negative regulator of PDH [18], and downregulates the expression of PARK2, which activates PDH [19]. The combination of both effects in *TP53* mutants produces a decreased PDH activity and consequently a lower flux of pyruvate to acetyl-CoA [20], while increasing lactate production. In addition, *TP53* mutants show a decrease in *SCO2* induction, which normal expression completes the electron transport chain [17]. Thus, the mutation in *TP53* can be correlated with the downregulation of the OxPhos metabolism. The lower derived mitochondrial activity explains the reduced consumption of glutamine, BCAAs, and serine compared to RT4 cells and thus the high weight that glucose has on their diet.

Although glucose is the almost exclusive nutrient and lactate is the primary excretion product of 5637 cells, we did not find signs of upregulation of the glucose uptake. The almost identical values of its consumption rate of the three cell lines, which show different proliferative capacities, would indicate that there is no activation or over-expression of the glucose transporters. There is a precedent that supports our hypothesis: the expression of GLUT1 and GLUT4 was lower or invariant between RT4 and TCCSUP cells, although the latter excreted more lactate [7]. These results contrast with the known increase in the expression of these receptors caused by the alteration in *TP53* under hypoxic conditions [20]. Our results suggest that this does not occur in conditions of normoxia, and that the greater use that these cells make of glycolysis is due to a downregulation of OxPhos.

The genomic signature of RT4 cells shows a fusion between *FGFR3* and *TACC3* genes, which is called *F3-T3* [6,21]. It was not until very recently that the connection between this genomic alteration and cancer metabolism was proposed [21]. The authors have observed that glioblastoma tumors harboring the *F3-T3* cluster showed an activation of mitochondrial functions, specifically OxPhos and mitochondrial biogenesis. The anabolic pathways related to this activity lead to an increase in Reactive Oxygen Species (ROS) concentration, stimulating mitochondrial respiration and tumor growth [21]. This could explain the activated OxPhos metabolism that we have found in the RT4 cells, which determines a higher consumption of glutamine, BCAAs, and serine. Both glutamine and BCAAs can act as anaplerosis metabolites, driving the TCA cycle through the generation of alpha-ketoglutarate [22] and acetyl-CoA, respectively [23]. Likewise, the increased uptake and catabolism of serine, with a higher excretion of formate and glycine, is related to a higher mitochondrial activity. In vitro experiments have shown that the requirements for the increased excretion of formate are a sufficient availability of serine, active OxPhos metabolism, and competent mitochondrial one-carbon metabolism [13,24]. The high concentration of formate promotes cell invasion through a mechanism that has yet to be elucidated [12].

In the case of the RT112 cells, the coexistence of alterations in *FGFR3* and *TP53* provide them with a mixed metabolic character. The mutation in *TP53* results in a higher weight of lactate in the excretion profile than that of RT4 cells. *F3-T3* fusion results in higher mitochondrial activity than 5637 cells, which in turn increases the consumption of glutamine and BCAAs. However, the lower activity of OxPhos with respect to RT4 cells, due to the decreased activity of PDH and the lower expression of *SCO2*, has an effect on the serine’s catabolism, explaining the lower excretion of formate and glycine. This makes these cells an interesting test bed to measure the effect of two co-existing genotypic alterations with opposite consequences on the phenotype.

The metabolic diversity that we have found between these cell lines is clearly reflected in the dependence on glucose or glutamine for growth that they have shown in a previous experiment. 5637 cells do not grow without glucose in the medium, but their proliferation is normal without glutamine [25], which is in line with an almost exclusive use of glycolysis. Surprisingly, RT4 cells grow normally in the absence of glucose, but the lack of glutamine completely stops their growth [25], even though under normal conditions, we have observed that glucose represents 68% of their diet and glutamine represents only 13%. This is a significant example of the extreme metabolic plasticity of these cancer cells, which may be the consequence of the proposed decoupling between glycolysis and OxPhos in tumor cells [26]. It was suggested that this mechanism allows these cells to use glutamine in unconventional ways, such as traveling in reverse through the flow of TCA to feed the biosynthesis of fatty acids, consenting it to completely replace glucose. Although there are no data on the growth of RT112 cells based on the availability of glucose and glutamine, a cell line with a similar genotype, SW780 [6], showed a mixed behavior: they need both nutrients to grow normally [25].

We are presenting the first evidence that OxPhos can significantly contribute to the metabolism of certain bladder cancer cells. The upregulation of this state was generically observed in tissue extracts from UBC patients [27], but it was not clear what role this anaplerotic activity might play. Most of the metabolic studies on UBC have emphasized the hypoxic conditions in which most tumors grow, with the consequent dramatic increase in the rate of glycolysis. However, how much BC mitochondria contribute to ATP production and critical biomass synthesis vs. the degree to which glycolysis and other enzymatic pathways perform this function was not clear [28]. Still, recent studies have shown that a relatively high number of tumors show comparable or even higher rates of glucose processed by OxPhos than those seen in normal tissues [29,30]. This led to the proposal that there are two classes of cancer metabolism [12]: one with a low oxidative metabolism, which in the context of UBC would be represented by *TP53* altered cells, and another with an active oxidative metabolism coupled to normal mitochondrial function, which is represented by cells with the *FGFR3* genomic signature. A further validation of our metabolic analysis could be achieved by altering the balance between OxPhos and glycolysis with pharmacological inhibitors or by silencing genes involved in these pathways. In addition, the activity of the enzymes responsible for these metabolic changes can be measured to further sustain these observations. All these experiments are in progress and will be the subject of future studies.

However, it is important to remember that cell lines often do not accurately reflect the genetic and phenotypic diversity of primary tumors, because other important players, such as stromal and inflammatory components, are not represented in the experiments [6]. Moreover, it has been seen that in UBC tumors, there is a metabolic coupling between the catabolic stromal and cancer cell compartments, and the anabolic cancer cells [31]. What allows cancer cells to adapt faster than normal cells to changing conditions and to use different metabolic pathways, such as glycolysis and OxPhos, according to their needs? A recently developed theory states that unlike normal cells that possess separate oxidative and glycolytic states, cancer cells appear to have access to a hybrid state in which both metabolic modes coexist [32]. This hybrid state gives them increased metabolic plasticity compared to normal cells, and it may ultimately be the main cause that promotes tumorigenesis and metastasis. It is expected that UBCcls will use this mechanism to quickly adapt to the different microenvironments they find within tumors. However, the different phenotypes that they possess, constrain their responses, and can have a crucial influence on the direction that the progression of UBC finally takes.

## 4. Materials and Methods

### 4.1. Cell Culture

UBCcls were grown in 12 mL of RPMI containing 10% fetal bovine serum (FBS). In a typical experiment, cells were grown for 1, 2, and 3 days in separate T75 flaks. At the end of each period, 2 mL of medium were collected from the corresponding flaks. This allowed measurement of the variation in metabolite concentration after each time period using each cell culture as a time point reference for the next while maintaining a constant total amount of culture medium for each condition. A total of 4 to 5 replicates of these experiments were performed, obtaining a total of 38 samples. Samples of RPMI medium were collected under the same experimental conditions and for each time point. These samples were used in all cases as references to discriminate whether the variation in metabolite concentrations was due to the cellular metabolic action or to chemical instability or enzymatic action of the serum. Then, the collected samples were centrifuged at 1400 rpm for 5 min at 4 °C, and the supernatant solutions were stored at −80 °C until the NMR analysis.

### 4.2. Samples for NMR Analysis

Samples for NMR analysis were prepared by thawing and centrifuged at 4000 rpm for 10 min at 4 °C. First, 500 mL of the supernatant were added to 100 mL of phosphate buffer (K_2_HPO_4_/NaH_2_PO_4_ 640 mM, NaN_3_ 12.8 mM, pH 7.4, 10% D_2_O), which contained 14.4 mM of 3-(Trimethylsilyl) propionic-2,2,3,3-d_4_ acid (TSP) as the internal standard. The final solution was transferred to a 5 mm NMR tube.

The pH of each sample was carefully controlled throughout the experiment to exclude acidification due to lactate production, which could change metabolism during cell proliferation. The maximum pH change observed was 0.2 units, with an accuracy of 0.1, and it was considered constant.

### 4.3. Spectra Acquisition

NMR spectra were acquired with a Bruker Avance 700 MHz spectrometer (Billerica, MA, USA) equipped with a Triple resonance TXI probe and a SampleXpress Lite autosampler. Each spectrum was acquired using a noesypr1d pulse sequence for water suppression with saturation pulses before and during the mixing time and with the addition of a CPMG filter to reduce broad signals from proteins and lipids. Presaturation pulses contained in the sequence reduce the intensity of exchangeable protons such as NH or OH, but their integrals were not used to determine the absolute concentration of the metabolites. Experiments were performed at 298K, with 100 ms of mixing time, 14 ms of CPMG filter, 12 ppm of spectral width, 2 s of acquisition time, a relaxation delay of 3 s, and 128 transients. The total duration of each experiment was 1 h and 48 min. ^1^H-^1^H TOCSY experiment was acquired at 600 MHz with spectral windows of 13.0 and 10.4 ppm, with a carrier frequency at 4.7 ppm and using 4096 × 512 points and 8 transients. Water was suppressed using presaturation pulses. ^1^H-^13^C HSQC experiments were acquired with a spectral window of 15 ppm × 100 ppm (carrier frequencies at 4.85 and 46.5 ppm for the aliphatic region and 110 ppm for the aromatic region) using 2048 × 600 data points and 36 transients.

### 4.4. Spectral Analysis

The assignment of the resonances and the quantification of the concentration of the different metabolites was done using Chenomx NMR Suite 8.5. The use of the spectra database contained in this software allows performing a manual deconvolution of the different signals and thus determining the concentration of the compounds that form the mixture. The spectra were processed using a 0.5 Hz line broadening followed by manual phase and baseline corrections. For each spectrum, the linewidth of the signals was carefully measured to allow the best deconvolution fitting. The chemical shifts used in the assignment of the metabolites were corroborated using ^1^H-^1^H TOCSY and ^1^H-^13^C HSQC experiments.

### 4.5. Analysis of Metabolite Variations

We have calculated the daily consumption or excretion of all quantified metabolites, $\Delta {\left[M\right]}_{i}^{k}$, at three different time points: after 1, 2 or 3 days by using the following equation:

(1) $$\Delta {\left[M\right]}_{i}^{k}\;=\;{\left[M\right]}_{i}^{k}-\;{\left[M\right]}_{i}^{k-1}$$

where ${\left[M\right]}_{i}^{k}$ refers to the concentration of the *i*-metabolite in the *k* sample, with *k* = 1, 2, or 3 indicating the total time of cell growth in days. The reference medium is codified by *k =* 0 and represents RPMI.

For the slope analysis to determine the lactate/alanine ratio, we have calculated the variations with respect to the culture medium:

(2) $$\Delta {\left[M\right]}_{i}^{k}\;=\;{\left[M\right]}_{i}^{k}-\;{\left[M\right]}_{i}^{0}.$$

### 4.6. Normalization of Metabolite Variations

Variations in metabolite concentrations in the extracellular medium depend, in addition to intrinsic metabolism, on the number of cells contributing to consumption or excretion. Therefore, it is necessary to obtain a normalization of the data when working with cell lines that grow at different rates. This can be done by calculating the variation per cell, considering the ratio between the rate of consumption or excretion and the rate of cell proliferation. In this work, we have applied a second type of normalization that uses the ratio between the variation of the concentration of a compound and the sum of all the concentration variations that show the same sign for $\Delta {\left[M\right]}_{i}^{k}$: positive for excretion and negative for consumption. This ratio defines the weight of a metabolite in the total consumption (the cellular diet) or total excretion and is independent of the number of contributing cells. For a detailed derivation of the mathematical equations used for the two normalization schemes, please refer to the Supplementary Material (S.M.) [33].

### 4.7. Statistical Analysis

The multivariate analysis of the data was carried out using SIMCA-P (version 15.0.2. Umetrics AB, Umea, Sweden). The classification model was built using Orthogonal PLS Modeling (OPLS) [34] in the discriminant version (OPLS-DA). The variables used were the different $q_{M_{i}^{k}}$ and $w_{M_{i}^{k}}$ calculated from the Equations (13) and (14) (S.M.). The robustness of the models was evaluated through the following parameters: R^2^Y, predicted percentage of the response; R^2^X, variation of X explained by the model and Q^2^, goodness of prediction. R^2^ varies between 0 and 1, and Q^2^ varies between −1 and 1.

A model is considered predictive when Q2 is greater than 0.5. The influence on Y variation of every variable, called Variable Importance in the Projection (VIP), was used to select those metabolites involved in class discrimination. Anova tests of the cross-validated residual (CV-ANOVA) were performed to check if the OPLS-DA model has a significantly smaller cross validated predictive residuals than just the variation around the global average. All these parameters were calculated using SIMCA-P.

## 5. Conclusions

Three UBC cell lines representative of low, intermediate, and high tumor progression risks were metabolically characterized by measuring nutrient intake and product excretion over three days. Our results indicate that the low risk of tumor progression is associated with an oxidative metabolic state of the cells, while the high risk of progression is associated with an elevated use of glycolysis. These different metabolic states, and even an intermediate one, are a reflection of the different genotypes of the studied cells. Our observations represent the first evidence of UBC cell lines relying fundamentally on an active OxPhos, which is caused by an *FGFR3* alteration. The cells related to a high risk of progression and mutation in *TP53* depend more on glycolysis, but in the presence of sufficient quantities of oxygen, this higher non-oxidative activity is the consequence of a downregulation of OxPhos and not of an activation of glucose uptake. We can envisage that these preliminary observations can be translated in the future, after in vivo validation, into clinical practice, and they could also open new avenues to better understand the mechanisms driving cancer progression and to identify potential biomarkers for an effective and affordable monitoring of this neoplasia.

## Supplementary Materials

Supplementary Materials can be found at https://www.mdpi.com/1422-0067/21/21/8107/s1.
