# Supplementary material for: The Interplay between Oxidative Phosphorylation and Glycolysis as a Potential Marker of Bladder Cancer Progression
Source: Int J Mol Sci. 2020 Oct 30;21(21):8107. doi: 10.3390/ijms21218107 (PMC7662640; doi:10.3390/ijms21218107)
Supplement: Supplementary file 1 [file ijms-21-08107-s001.pdf]

Normalization of metabolite variations

Absolute metabolite variations depend on the number of cells contributing to it during the time. We have applied two ways of normalizing this variation: (i) by expressing the variation per cell, considering the ratio between the rate of variation and the rate of cell proliferation; (ii) using a ratio between the metabolite variation and the sum of all concentrations of metabolites showing the same sign for $\Delta[M]_i^k$: positive for excretion and negative for consumption. This ratio defines the weight of the metabolite on the total consumption (the cellular diet) or the total excretion and is independent from the number of contributing cells.

- ***Variation per cell:***

Cell number variation is given by the product between the specific growth rate m and the number of cells X:

$$r_X = \mu X \tag{2}$$

One of the most popular models to describe cell growth is that of Monod [1], that links m to the concentration of the nutrient S used by the cells.

$$\mu = \frac{\mu_{max}[S]}{K_S+[S]} \tag{3}$$

where m_{max} is the maximum growth rate and K_S the concentration of substrate at which a $m_{max}/2$ rate is achieved. In our experimental conditions, main metabolites for cell growth are glucose and glutamine, and their concentrations can be safely considered much higher than K_S. In this condition, the specific rate can be considered constant during the entire experiment with cells growing at the maximum rate:

$$\mu = \mu_{max} \tag{4}$$

The mass/energy of a substrate that is consumed by an organism goes towards two uses: maintenance of the cell, independent of growth, and production of new cellular components, which ultimately become new cells. The rate of substrate consumed in order to maintain the cells is proportional to the number of cells:

$$-\frac{\delta S}{\delta t} = m_S X \tag{5}$$

where m_S is the maintenance coefficient, with units pmol substrate/(cell*h)

The rate of substrate consumed to produce more cells is proportional to the rate of new cells produced:

$$-\frac{\delta S}{\delta t} = \frac{1}{Y_{X/S}}\frac{\delta X}{\delta t} = \frac{r_X}{Y_{X/S}} \tag{6}$$

where $Y_{X/S}$ is the cell yield coefficient and represents the number of cells formed per pmol of substrate consumed. The total substrate utilization may be written:

$$-r_S = -\frac{\delta S}{\delta t} = m_S X + \frac{r_X}{Y_{X/S}} \quad (7)$$

Using equation [2] and condition [4] we can rewrite equation [7] into:

$$-r_S = -\frac{\delta S}{\delta t} = \frac{m_S r_X}{\mu_{max}} + \frac{r_X}{Y_{X/S}} = \left(\frac{m_S}{\mu_{max}} + \frac{1}{Y_{X/S}}\right) r_X \quad (8)$$

We can group all the constants into a single quantity, q_S, which will represent the specific rate of substrate consumption with units pmol substrate/cell, and incorporates both the maintenance and growth needs of the cells:

$$q_S = -\left[\frac{m_S}{\mu_{max}} + \frac{1}{Y_{X/S}}\right] = \frac{r_S}{r_X} \quad (9)$$

We can approximate the slope values by the variations in metabolite concentration day by day:

$$\frac{\delta S}{\delta t} \approx \frac{\Delta[S]_i^k}{\Delta t} \quad (10)$$

where $\Delta[S]_i^k$ has the same meaning as in equation [1] but refers to a consumed metabolite. A similar approximation can be used to estimate r_X:

$$r_X \approx \frac{X^k - X^{k-1}}{\Delta t} = \frac{\Delta[X]^k}{\Delta t} \quad (11)$$

As we calculate the ratio between r_S and r_X, Dt is simplified, giving the following expressions for a substrate (S) or product (P)

$$q_{S_i^k} = \frac{\Delta[S]_i^k}{\Delta[X]^k} \qquad q_{P_i^k} = \frac{\Delta[P]_i^k}{\Delta[X]^k} \quad (12)$$

With $q_{S_i^k} < 0$ and $q_{P_i^k} > 0$.

- ***Variation per composition:***

We can also normalize the daily variations by calculating the relative weight that the *i*-metabolite variation has on the total consumption ($w_{S_i^k}$)or excretion ($w_{P_i^k}$) during the *k*-day. To do that, we calculate separately the sum of the variations of the *n* consumed and the *m* excreted metabolites and use the following equations:

$$w_{S_i^k} = -\frac{\Delta[S]_i^k}{\sum_{j=1}^{n} \Delta[S]_j^k} \qquad w_{P_i^k} = \frac{\Delta[P]_i^k}{\sum_{l=1}^{m} \Delta[P]_l^k} \quad [13]$$

where the negative sign in the left expression is needed to distinguish weights on the diet from those on the excretion and allowing $w_{S_i^k}$ to have the same sign as $q_{S_i^k}$.

References

[1] Monod J. The growth of bacterial cultures. *Ann Rev Microbiol.* 1949;3:371-394.

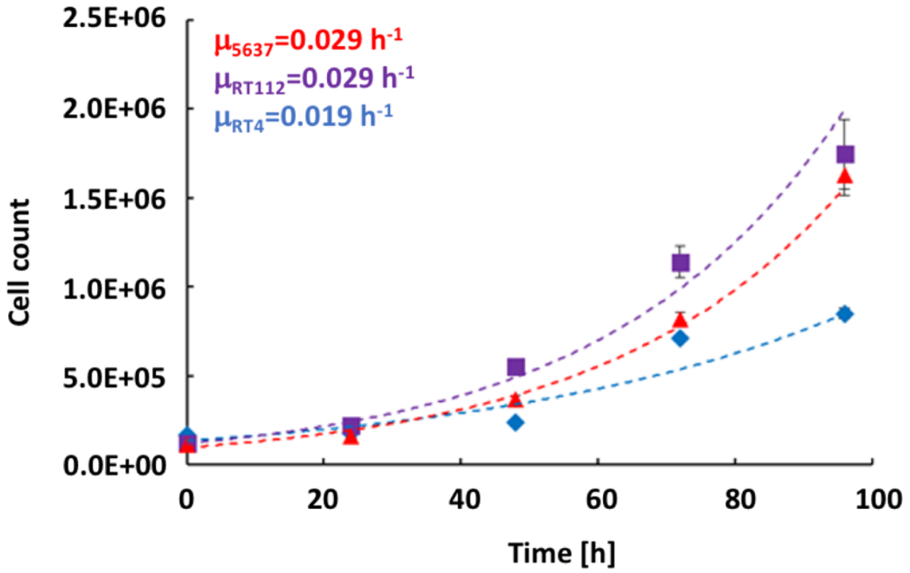

Figure S1. Cell proliferation curves for the three UBC cell lines and the respectively calculated intrinsic growth rates by best fit of the experimental points.

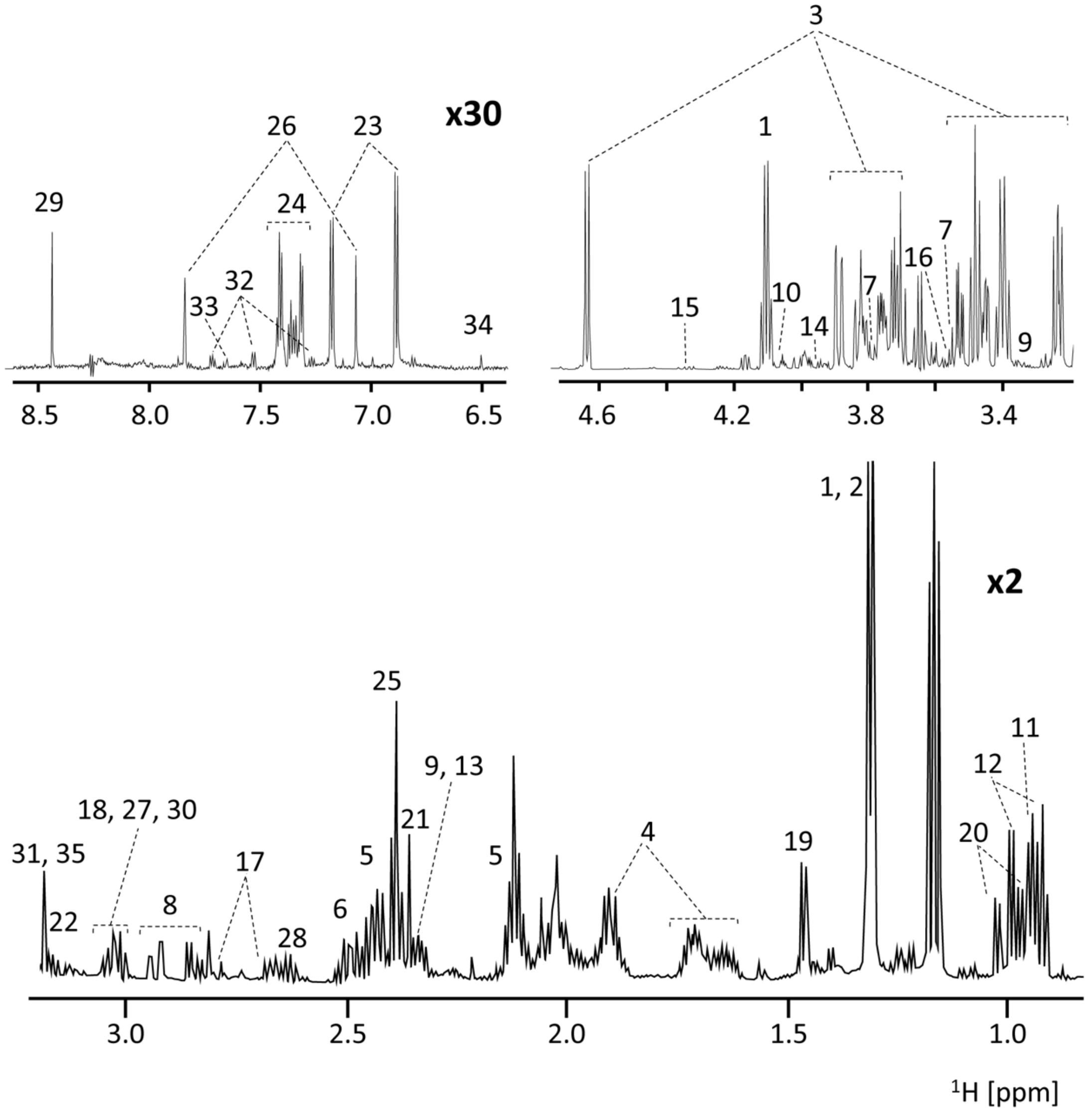

Figure S2. Representative 700 MHz ^{1}H-NMR spectrum of RPMI medium after 2 days of 5637 cell growth. 1: Lactate; 2: Threonine; 3: Glucose; 4: Arginine; 5: Glutamine; 6: Pyroglutamate; 7: Fructose; 8: Asparagine; 9: Proline; 10: myo-Inositol; 11: Leucine; 12: Isoleucine; 13: Glutamate; 14: Serine; 15: t-4-OH-proline; 16: Glycine; 17: Aspartate; 18: Lysine; 19: Alanine; 20: Valine; 21: Pyruvate; 22: Cystine; 23: Tyrosine; 24: Phenylalanine; 25: Succinate; 26: Histidine; 27: Ornithine; 28: Methionine; 29: Formate; 30: Creatinine; 31: Choline; 32: Tryptophan; 33: Pyridoxine; 34: Fumarate; 35: Acetylcholine.

Table S1. [1]H and ^{13}C assignments of metabolites identified in extra-cellular medium. [1]H and ^{13}C chemical shifts reported with respect to the TSP signal.

	[1]H n (ppm)	[13]C n (ppm)
**Alanine**		
CH_3	1.468	19.1
CH-a	3.778	53.4
**Arginine**		
CH-a	3.758	57.1
CH_2-b	1.919	30.3
CH_2-g	1.681	26.7
CH_2-d	3.233	43.4
**Asparagine**		
CH-	3.982	54.0
CH_2-	2.871, 2.930	37.4
**Aspartate**		
CH_2-	2.671, 2.799	
**Choline**		
CH_3	3.179	56.5
N-CH_2	3.508	70.2
**Creatinine**		
CH_3	3.02	32.9
CH_2	4.03	59.0
**Cystine**		
CH-	4.089	
CH_2-b	3.174, 3.369	
**Formate**		
CH	8.451	173.8
**Fructose**		
aCH-1	4.105	78.1
bCH-1	3.789	70.2
aCH -2	4.106	77.7
bCH-2	3.879	72.4
bCH-3	3.992	71.8
**Fumarate**		
CH	6.509	
**Glucose**		
aCH -1	5.227	95.1
bCH-1	4.606	98.4
aCH -2	3.53	74.2
bCH-2	3.238	77.0
aCH -3	3.697	75.6
bCH-3	3.469	78.7
aCH -4	3.403	72.5
bCH-4	3.403	72.5
aCH -5	3.808	74.1
bCH-5	3.469	78.7
aCH_2-6	3.828	63.5
bCH_2-6	3.732, 3.887	63.6
**Glutamate**		
CH-	3.731	57.5
CH_2-	2.087	30.0
CH_2-	2.339	36.2
**Glutamine**		
CH-	3.749	57.4
CH_2-	2.123	29.3
CH_2-	2.449	33.6
**Glycine**		
CH_2	3.551	44.4
**Histidine**		
CH-	3.990	57.3
CH_2-	3.256, 3.297	30.2
CH- 2	7.122	120.0
CH-e1	7.967	138.7
**Isoleucine**		
CH_3-d	0.933	13.9
CH_3-g2	0.997	17.4
**Lactate**		
CH_3	1.317	22.9
CH	4.106	71.3
**Leucine**		
CH_3-d1	0.948	23.7
CH_3-d2	0.958	24.7
**Lysine**		
CH-	3.767	57.0
CH_2-	1.899	32.7
CH_2-	1.426, 1.488	24.2
CH_2-	1.716	29.2
CH_2-e	3.015	42.0
**Methionine**		
CH-	3.847	
CH_2-	2.186, 2.109	
CH_2-	2.631	
CH3-e	2.126	
**Myo-inositol**		
CH-1	4.059	75.0
CH-2	3.628	75.1
CH-4	3.282	77.2
**O-acetylcholine**		
N-CH_3	3.209	
**Ornithine**		
CH_2-	3.046	
**Phenylalanine**		
CH-	7.319	
CH-e	7.419	
CH-z	7.367	
**Proline**		
CH_2-	3.327	
**Pyridoxine**		
CH-6	7.653	
**Pyroglutamate**		
CH-	4.169	61.2
CH_2-	2.025, 2.496	28.0
CH_2-	2.393	32.4
**Pyruvate**		
CH_3	2.364	29.2
**Serine**		
CH-	3.84	59.2
CH_2-	3.956, 3.979	63.0
**Succinate**		
CH_2	2.397	36.8
**Threonine**		
CH_3-g	1.317	22.2
CH-	4.254	68.7
CH-	3.588	63.2
**trans-4-Hydroxy-L-proline**		
CH-	4.326	62.4
**Tryptophan**		
CH-	4.048	57.8
CH_2-	3.292, 3.473	29.2
CH-1	7.71	121.2
CH-2	7.523	114.7
CH-3	7.307	128.0
CH-4	7.263	125.2

CH-5	7.176	122.2
**Tyrosine**		
CH-	7.175	133.5
CH-e	6.862	119.1
**Valine**		
CH_3-g1	1.038	20.7
CH_3-g2	0.984	19.5
CH-b	2.251	31.8
CH-a	3.606	63.2

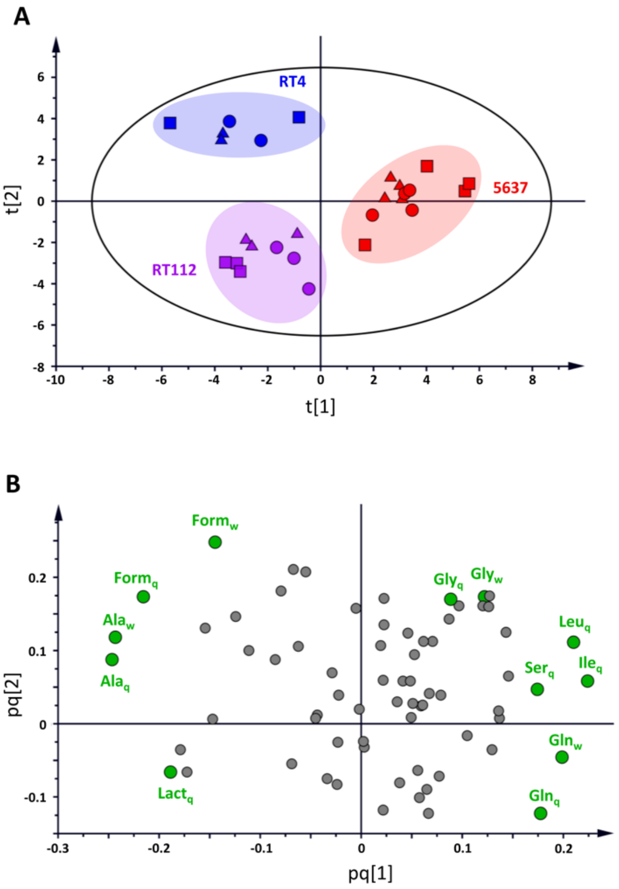

Figure S3. (**A**) Score plot of the OPLS-DA Model calculated with the sum of all daily variations and using the two normalization methods. The model has a good predictive power (Q^2=0.868) and is highly significant (CV-ANOVA=8.0E-05). (**B**) Loading plot showing the variables that mostly contribute to the separation of exo-metabolomes from the three cell lines (in green). The suffix *w* denotes normalization by diet or excretion profile, while *q* indicates normalization by cell growth. Abbreviations used: Ala: alanine, Form: formate, Gly: glycine, Gln: glutamine, Leu: leucine, Ser: serine.
